# Supplementary material for: Learning curve and surgical outcome of robotic assisted colorectal surgery with ERAS program
Source: Sci Rep. 2022 Nov 29;12:20566. doi: 10.1038/s41598-022-24665-w (PMC9709162; doi:10.1038/s41598-022-24665-w)
Supplement: Supplementary file 1 — Supplementary Information 1. [file 41598_2022_24665_MOESM1_ESM.zip › LSC Raw data-Ñ[▒K/090/P_ñw╝╨░O▒Kñσ.pdf]

[redacted]

Pathologic diagnosis:
1. Rectum, LAPA anterior resection ---
a. Status post pre-surgical therapy, with deep ulcer, xanthomatous inflammation and organizing granulation tissue reaction. No residual tumor present. The bilateral cut ends and perirectal lymph nodes (0/13) are free of tumor.
b. Fibrinoid necrosis, thrombosis and fibrointimal hyperplasia of small arteries, multiple. See Note.
2. IMA root, dissection --- Unremarkable fibroadipose tissue.
3. Colon, cut end, distal, resection --- Unremarkable.

Ancillary study for diagnosis:
IHC stains for AE1/AE3 (no residual tumor), and b-catenin (- on target lesion) done on section J.

Prognostic and predictive factor:
1. Tumor size: No residual tumor.
2. Depth of invasion: No residual tumor.
3. Histologic grade: No residual tumor.
4. Angiolymphatic invasion: Not identified.
5. Perineural invasion: Not identified.
6. Tumor deposits (discontinuous extramural extension): Not identified.
7. Macroscopic tumor perforation: Not identified.
8. Surgical Margins: a) Proximal Margin: Uninvolved by invasive carcinoma. b) Distal Margin: Uninvolved by invasive carcinoma. c) Radial or Mesenteric Margin: Uninvolved by invasive carcinoma.
9. Lymph node status: Uninvolved (0/13).
10. Extranodal involvement: Not identified.
11. Tumor regression grading S/P CCRT: Present, no viable cancer cells (complete response, score 0).
12. Type of polyp in which invasive carcinoma arose: Not identified.
13. Additional pathologic findings: Fibrinoid necrosis, thrombosis and fibrointimal hyperplasia of small arteries, multiple. See Note.
14. Pathological TNM Stage: ypT0N0(According to the eighth edition, American Joint Committee on Cancer Staging Guidelines for Tumors).
15. TNM descriptors: y (Post-treatment).

Gross description:
The specimen consists of 1) a segment of rectum, 13cm in length and 3 cm in proximal cut end circumference, 2.5 cm in distal circumference, and perirectal soft tissue. A gray tan firm annular elevated and centrally ulcerative tumor, 3x2.6 cm, is noted, 3 cm to the nearest cut end. On cross section, tumor is near to CFM. The both cut ends are free of tumor grossly. 2) IMA root, a piece of tan yellow soft tissue, 4x2.5x0.5 cm. 3) distal cut end, a piece of tan mucosa-covered soft tissue, 3x2x1 cm. All for section: A-B) specimen 2 C) specimen 3; Representative part for section: D-E) both cut ends F-H) tumor and CFM J) tumor and serosa K) tumor and normal L-M) lymph nodes.

Note:
The histology finding of vascular change, including fibrinoid necrosis, thrombosis and fibrointimal hyperplasia of small arteries, is suspicious for thrombotic microangiopathy. Clinical correlation, survey and follow up are recommended.
#T-59300_2 #M-38000_2 0 1105 SP0000
#T-C4560_2 #M-09183_2 0 1105 000000

已電子簽章

敬告：本單僅供醫師參考使用，不具任何申請效力！！

請病患妥為保管，若不慎遺失需自負個人資料外洩責任！
